# *In Vitro* Characterization and Concerted Function of Three Core Enzymes of a Glycyl Radical Enzyme - Associated Bacterial Microcompartment

**DOI:** 10.1038/srep42757

**Published:** 2017-02-16

**Authors:** Jan Zarzycki, Markus Sutter, Niña Socorro Cortina, Tobias J. Erb, Cheryl A. Kerfeld

**Affiliations:** 1 Max-Planck-Institute for Terrestrial Microbiology, Karl-von-Frisch-Str. 10, D-35043, Marburg, Germany; 2 MSU-DOE Plant Research Laboratory, Michigan State University, 612 Wilson Road, East Lansing, MI 48824, USA; 3 Molecular Biophysics and Integrated Bioimaging Division, Lawrence Berkeley National Laboratory, 1 Cyclotron Road, Berkeley, CA 94720, USA; 4 Department of Biochemistry & Molecular Biology, Michigan State University, 603 Wilson Road, East Lansing, MI 48824, USA; 5 Berkeley Synthetic Biology Institute, Berkeley, CA, USA; 6 Department of Plant and Microbial Biology, University of California, 111 Koshland Hall, Berkeley, CA 94720, USA

## Abstract

Many bacteria encode proteinaceous bacterial microcompartments (BMCs) that encapsulate sequential enzymatic reactions of diverse metabolic pathways. Well-characterized BMCs include carboxysomes for CO_2_-fixation, and propanediol- and ethanolamine-utilizing microcompartments that contain B_12_-dependent enzymes. Genes required to form BMCs are typically organized in gene clusters, which promoted their distribution across phyla by horizontal gene transfer. Recently, BMCs associated with glycyl radical enzymes (GREs) were discovered; these are widespread and comprise at least three functionally distinct types. Previously, we predicted one type of these GRE-associated microcompartments (GRMs) represents a B_12_-independent propanediol-utilizing BMC. Here we functionally and structurally characterize enzymes of the GRM of *Rhodopseudomonas palustris* BisB18 and demonstrate their concerted function *in vitro*. The GRM signature enzyme, the GRE, is a dedicated 1,2-propanediol dehydratase with a new type of intramolecular encapsulation peptide. It forms a complex with its activating enzyme and, in conjunction with an aldehyde dehydrogenase, converts 1,2-propanediol to propionyl-CoA. Notably, homologous GRMs are also encoded in pathogenic *Escherichia coli* strains. Our high-resolution crystal structures of the aldehyde dehydrogenase lead to a revised reaction mechanism. The successful *in vitro* reconstitution of a part of the GRM metabolism provides insights into the metabolic function and steps in the assembly of this BMC.

Bacterial microcompartments (BMCs) are polyhedral organelles found in a wide range of phyla^1^. BMCs are subcellular nanoreactors that typically encapsulate several enzymes catalyzing sequential steps of metabolic pathways. In contrast to membrane-delimited organelles, selective permeability of BMCs is conferred by a proteinaceous shell, which allows small polar and charged metabolites to pass, and appears to exclude passage of uncharged, non-polar molecules.

The majority of BMCs are involved in catabolism^1^,^2^. The general model^2^,^3^ for the biochemical transformations within these ‘metabolosomes’ was derived from experimental characterization of a few catabolic BMCs^3^,^4^,^5^,^6^ and bioinformatic analysis^1^,^7^ (Fig. 1). Catabolic BMCs typically generate toxic and/or volatile aldehyde intermediates through the action of signature enzymes, which differ among functionally distinct BMCs. The subsequent conversion of the aldehyde is catalyzed by the other core enzymes: an acylating aldehyde dehydrogenase, an alcohol dehydrogenase, a phosphotransacylase and a carboxylic acid kinase^1^,^2^ (Fig. 1).

A large and distinct group of the catabolic BMCs was recently discovered^1^. These BMCs contain glycyl radical enzymes (GREs) and their cognate activating enzymes (AEs) as signature enzymes. GRE-associated microcompartments (GRMs) appear to be diverse: at least three functionally different types were predicted by bioinformatic analysis^1^,^7^. GREs that function as choline trimethylamine-lyases^8^,^9^,^10^,^11^,^12^, producing trimethylamine (TMA) and acetaldehyde, are found associated with two microcompartment subtypes, GRM1 & GRM2 (nomenclature according to Zarzycki J. *et al*.^7^). Another type of GRM (GRM5) seems to function in fuculose metabolism; the corresponding genes are expressed during the anaerobic degradation of fuculose^6^. GRM5 was proposed to employ two additional enzymes, a fuculose phosphate aldolase and a lactaldehyde reductase. Fuculose phosphate can be cleaved into dihydroxyacetone phosphate and lactaldehyde. The latter would then be reduced to 1,2-propanediol, hence the associated GRE was proposed to function as a 1,2-propanediol dehydratase^6^. Indeed the GRE of a GRM5 from *Roseburia inulinivorans* has very recently been crystallized and shown to function as 1,2-propanediol dehydratase^13^, as was proposed for this type of GRM^1^,^6^,^7^.

A far more widespread subtype of the GRMs (GRM3), found in α-proteobacteria, γ-proteobacteria, and firmicutes, was predicted to contain a GRE that also functions as a propanediol dehydratase^1^,^7^, but only shows 52% amino acid sequence identity to the GRM5 enzyme from *R. inulinivorans*. Here we systematically reconstruct a part of the metabolism of the GRM3 of *Rhodopseudomonas palustris* BisB18 by functionally and structurally characterizing three core enzymes, and by reconstituting the reaction sequence *in vitro*. We demonstrate that the GRE of *Rps. palustris* functions as a specific 1,2-propanediol dehydratase. Moreover, this signature enzyme is, to our knowledge, the first identified with an unusual intra-domain encapsulation peptide (EP) for packaging into the BMC. The *Rps. palustris* GRE binds to its AE, suggesting how the catalytic core of the organelle is assembled. Furthermore, we show that the acylating aldehyde dehydrogenase (AldDH), encoded within the GRM gene cluster, preferentially uses propionaldehyde as substrate. Moreover, we provide insights into the catalytic mechanism of AldDHs, by describing an acylated enzyme intermediate crystal structure, as well as clearly resolving the binding of CoA and propionyl-CoA in an acylating AldDH. By reconstitution of the core metabolism of the GRM3, we show that it functions analogously to the canonical PDU-BMCs, which employ B_12_-dependent enzymes. This is the only known example of functional redundancy among distinct types of BMCs. Moreover, our results confirm that the general metabolic model for catabolic BMC function (Fig. 1A) also applies to the newly identified group of GRMs (Fig. 1B), the largest and most widespread family of BMCs detected to-date.

## Results

### Structural and Functional Analysis of the GRM3-associated Aldehyde deyhdrogenase (AldDH)

After correcting the start site for the AldDH gene (see Methods), we found (using the criteria of Kinney J. N. *et al*.^14^) that the N-terminal 75 amino acids likely function as an EP^7^,^15^. The predicted EP of the AldDH comprises two amphipathic α-helices, each 12–15 residues, separated by 20–25 unordered residues. The second helix connects to the enzymatic domain by another ~20 amino acid, presumably flexible, linker^7^. Most of the experimentally identified or bioinformatically assigned EPs consist of one amphipathic α-helix^15^.

Two different conditions were used to crystallize the AldDH with propionyl-CoA. At pH 8.1, AldDH crystallized in the monoclinic space group *P* 2_1_ with one tetramer per asymmetric unit (ASU). A tetrameric state was also reported for the AldDH homolog of *C. phytofermentans*^16^. This is consistent with our gel filtration analysis where AldDH eluted with an apparent mass of 280 ± 15 kDa (55.5 kDa per monomer), the slightly higher molecular mass could be due to the flexible N-termini. The AldDH crystals diffracted to a resolution of 1.9 Å and the structure was solved by molecular replacement. The resulting model (PDB 5JFN) included all residues of the catalytic domains, as well as CoA or propionyl-CoA bound in the active site (Fig. 2A). No density was observed for the first 85 N-terminal residues comprising the putative EP and the Strep-tag. The only exception is a short unordered stretch of seven residues between the two α-helices of the putative EP in one of the four chains, which is involved in crystal contacts. The resolved N-terminus of each of the four subunits each point in different directions, suggesting there is no interactions among the EPs.

Interestingly, we observed additional electron density around a cysteine residue in each of the putative active sites, which indicated a covalent modification. This cysteine residue was shown to be involved in catalysis and proposed to be acylated and deacylated in the course of the reaction^17^,^18^,^19^. Mutation of this cysteine into serine or alanine results in a loss of catalytic activity^16^,^20^,^21^. Because the enzyme was crystallized in the presence of propionyl-CoA at pH 8.1, we assumed that the corresponding cysteine residue was propionylated and modeled this residue as S-propionylcysteine. The covalent modification fit well into the electron density (Fig. 2B,C), to our knowledge the only acyl-enzyme thioester intermediate captured in a crystal structure of an acylating AldDH (pfam00171) to date. In three of the four active sites of the tetramer we also found coenzyme A bound in close proximity to the modified cysteine (Fig. 2B,C). In the fourth active site we were able to fit propionyl-CoA, which probably resulted from an additional soaking step before freezing the crystals.

When AldDH was crystallized at pH 4.8 with propionyl-CoA, we obtained crystals with triclinic *P* 1 symmetry, diffracting to a resolution of 2.5 Å. The resulting model (PDB 5JFM) comprised two tetramers in the ASU, all chains lack electron density for the putative EPs. In the active sites we found electron density that allowed the fitting of either propionyl-CoA or CoA (Fig. S1A). Modification of the active site cysteine residue was not as apparent in this crystal form; this is likely due to the low pH, inhibiting the formation of a thiolate intermediate form of the cysteine^20^.

We used the same condition to co-crystallize AldDH with NAD^+^. These crystals had monoclinic P2_1_ symmetry, diffracted to a resolution of 2.3 Å and exhibited twinning (twin fraction of 37%). The resulting model (PDB 5JFL) comprises two tetramers in the ASU, again all chains lacking the putative EPs. Electron density in the active sites allowed the fitting of NAD^+^ (Fig. S1B). Interestingly, both co-substrates, NAD^+^ and (propionyl-)CoA, occupy a similar position in the two structures (Fig. S2) but with their adenine rings pointing in opposite directions. This was also reported for crystal structures of the AldDH homolog from *C. phytofermentans*^16^, and is consistent with a proposed bi-uni-uni-uni ping pong mechanism^16^,^17^,^18^,^19^, in which the aldehyde would bind to the active site together with NAD^+^. Subsequently NADH is released from the active site before CoA can bind and the final product, acyl-CoA, is released again^16^,^22^. In contrast to the AldDH structure from *C. phytofermentans* we were able to resolve the pantotheine moiety of the bound CoA as well as the propionyl thioester part of propionyl-CoA. Interestingly, we observed that the S-propionylcysteine assumes a different rotamer conformation than the non-acylated cysteine; this has implications for the reaction mechanism. The observed rotation prevents the interaction of the CoA with a conserved glutamate residue that was recently proposed to be responsible for the deprotonation of the CoA thiol^16^. The rotation of the S-propionylcysteine also opens up space for a proton transfer relay that could serve in the deprotonation of the CoA thiol (Fig. 3).

The proposed proton transfer relay involves Thr450 and His449, as well as two ordered water molecules. His449 is locked in the optimal tautomeric form for proton abstraction from the CoA thiol by hydrogen bonding to Thr450 (Figs 3 and 4). The corresponding histidine in the *C. phytofermentans* AldDH was previously proposed to catalyze the deprotonation of the catalytic cysteine residue^16^. We therefore suggest that this residue functions in two proton abstractions: from the catalytic cysteine (Cys330) and from the CoA thiol (Fig. 4). Both His449 and Thr450 are conserved among AldDHs, although the threonine is occasionally replaced by a serine (as in the *C. phytofermentans* AldDH). The overall reaction mechanism is discussed below. Data collection and refinement statistics are provided in Table 1.

To corroborate the hypothesis that the GRM3 of *Rps. palustris* functions in propanediol metabolism and produces propionaldehyde as an intermediate, we biochemically characterized the AldDH. We determined its kinetic parameters for propionaldehyde and acetaldehyde. The enzyme exhibited a higher maximal specific activity with propionaldehyde (*V*_max_ = 242 ± 14 μmol mg^−1 ^min^−1^) than with acetaldehyde (*V*_max_ = 117 ± 8 μmol mg^−1 ^min^−1^). Furthermore, the apparent *K*_m_-value for propionaldehyde was about four times lower than for acetaldehyde (1.2 ± 0.17 mM and 4.1 ± 0.7 mM, respectively). These values translate into a catalytic efficiency (*k*_cat_/*K*_m_) that is almost one order of magnitude higher with propionaldehyde (745.8 × 10^3^ M^−1 ^s^−1^) compared to acetaldehyde (105.6 × 10^3^ M^−1 ^s^−1^). The enzyme also exhibited highest activity with propionyl-CoA when measuring the activity in the reverse direction (reduction of acetyl-CoA, propionyl-CoA, and 3-hydroxypropionyl-CoA) (Table 2).

### Characterization of the GRM3 Signature Enzymes: The GRE and its Activating enzyme (AE)

When we anaerobically expressed a His-tagged version of the activating enzyme (AE) in *E. coli* we noticed that the C-terminally His6-tagged AE eluted at very low imidazole concentrations (50 mM) from the Ni-NTA resin, suggesting that the His-tag was not completely exposed. In order to determine if the AE contained [4Fe-4S] clusters, as predicted from the primary structure, the UV-VIS features of purified AE were analyzed in the absence and presence of DTT, dithionite, and oxygen. Moreover, we compared AE samples after [Fe-S] cluster reconstitution to untreated (*i.e.*, as purified) samples. Both, reconstituted and non-reconstituted samples, showed an absorbance band at around 400 nm, indicative of [4Fe-4S] clusters, whereas absorbance bands indicative of [2Fe-2S] or [3Fe-4S] clusters were not observed (Fig. 5). Reconstitution of the [Fe-S] cluster did not have a significant effect on the AE. This was evident by estimating the iron content in the protein based on the assumption that the extinction coefficient at 400 nm for the non-reduced [4Fe-4S] cluster is about 4,000 cm^−1 ^M^−1^ per iron atom^23^. For the reconstituted and the non-reconstituted AE, values of 13.1 and 12.3 iron atoms per monomer were determined. DTT (reduction potential of −330 mV) was not able to completely reduce the [Fe-S] clusters of the AE. A complete reduction was only achieved with dithionite (reduction potential of −660 mV) resulting in the loss of the UV-VIS features for the reconstituted and non-reconstituted samples. After exposing the reduced samples to ambient oxygen concentrations, the signal at 400 nm was recovered. However, after prolonged exposure to oxygen the signals were lost and the protein started to precipitate, underscoring the oxygen sensitivity of the AE.

A recombinant His-tagged version of the GRE formed inclusion bodies when produced anaerobically in *E. coli*. Only a small fraction of the protein could be purified in a soluble form, which started to precipitate moments after elution from the nickel-affinity column. BMC-associated enzymes are often insoluble due to the EP; its removal frequently results in the production of soluble protein^16^,^24^,^25^,^26^. EPs are required for BMC assembly^14^,^15^,^27^,^28^, but in contrast to other BMC-associated GREs the GRM3 type does not possess an obvious N- or C-terminal EP. Inspection of the primary structure of GRM3 GRE suggested that the EP was instead located intramolecularly^7^. Bioinformatic analysis predicted an insertion domain that comprises two α-helices (~15 residues each), which are connected to one another via a short loop (~5 residues) and are flanked by poorly conserved, unordered linkers (~15 residues on each side). One of the predicted helices shows the characteristic amphipathicity of EPs^7^. Interestingly, the expected topology of this EP is reminiscent of the putative EP of the GRM3 AldDH (see above and Zarzycki J. *et al*.^7^). The putative inter-domain EP of the GRM3 GRE is missing in the closely related B_12_-independent glycerol dehydratase^7^ (GDH) of *Clostridium butyricum*^22^,^29^. The primary structure of this enzyme is 55% identical to the *Rps. palustris* GRE, but is not associated with any BMC. We therefore designed a truncated version of the *Rps. palustris* GRE lacking the two putative intramolecular EP-helices (Fig. S3). Indeed, expression of the EP-less version of the GRE yielded more soluble protein. Although the purified enzyme still precipitated over time, this occurred more slowly (after several days at 4 °C) relative to the full-length enzyme. Interestingly, the predicted intramolecular EP of the GRM5 GRE^7^ from *R. inulinivorans* did not appear to cause precipitation in a recent study, but was, as noted by the authors, part of an oligomerization interface not previously observed in other GRE structures^13^. The amino acid sequences of the predicted intramolecular EPs of the GRM3 and GRM5 GREs are not strongly conserved and could therefore contribute to different characteristics in solution. However, the authors did not mention the GRE’s involvement in a microcompartment and did not acknowledge the possible of this additional inter-protein domain being an EP, and thus could not assign any function to it^13^.

Nevertheless, when we combined the *E. coli* cell pellets from truncated GRE and AE overexpressions to co-purify both enzymes via NTA-affinity chromatography, we noticed that the AE no longer eluted at low imidazole concentrations as observed before when it was purified in isolation. The AE now co-eluted with the GRE only at high imidazole concentrations. The AE was bound to the GRE in solution, indicative of a ‘piggy-back’ recruitment of the AE to the BMC by the GRE, which likely becomes encapsulated via the proposed intrinsic inter-domain EP.

Finally, to investigate the biochemistry of the GRE/AE complex, we examined whether the reductive cleavage of S-adenosylmethionine (SAM) during GRE activation resulted in the formation of 5′-deoxyadenosine (dA) or 5′-deoxy,5′-(methylthio)adenosine (MTA). In contrast to other SAM-dependent AEs, which produce dA, MTA formation had been reported for the AE of the GDH of *C. butyricum*^30^. In reconstituted GRM3 core reaction mixtures (see below) the formation of dA increased over time (Fig. S4). A background peak corresponding to the mass of MTA did not increase with time. Interestingly, the GRE of *Rps. palustris* shows a slightly higher percent identity in primary structure (55%) to the GDH of *C. butyricum* than to the 1,2-propanediol dehydratase from *R. inulinivorans* (52%). Despite all apparent similarities between the GRE and the AE of *Rps. palustris* as well as *R. inulinivorans* to their homologs in *C. butyricum* (GDH and its AE), there are distinct differences between the BMC-encapsulated and non-encapsulated enzymes with respect to structural motifs and biochemistry.

### Reconstitution of the GRM3 Core Reactions

To demonstrate the metabolic function of the GRM3 core, a mixture of purified, truncated GRE, AE and AldDH was incubated in the presence of dithionite, 1,2-propanediol, CoA, NAD^+^, and SAM. The mixtures were analyzed for the formation of propionyl-CoA by high resolution LC-MS. Propionyl-CoA was only detectable (Fig. 6A,B) when all (co-)substrates were present. No propionyl-CoA could be detected when 1,2-propanediol, NAD^+^, or SAM were omitted from the reaction mixture. In these experiments propionyl-CoA could only have been formed by the AldDH from propionaldehyde; this was produced by the truncated GRE, which had been activated by AE. Moreover, when we substituted propanediol with glycerol as a substrate, no formation of the corresponding CoA-thioester, 3-hydroxypropionyl-CoA (3OHP-CoA) was detected. Because the AldDH was able to use 3OHP-CoA at fairly high rates when tested alone (Table 2), this indicates that the GRE, and thus the GRM3, specifically function in 1,2-propanediol, and not glycerol, degradation.

## Discussion

**Figure 1:**
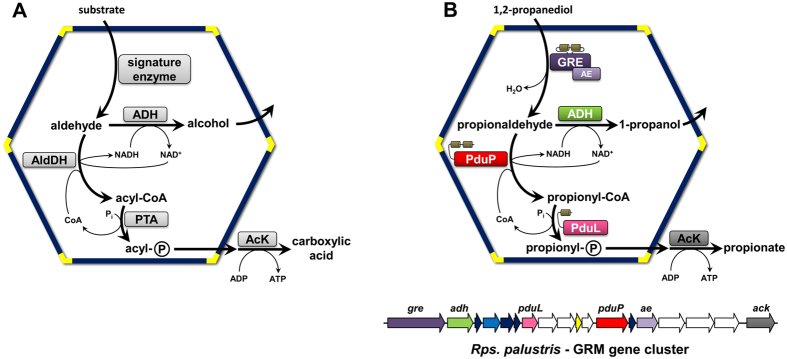
Schematics of pathways in catabolic bacterial microcompartments and the propanediol utilizing GRMs. (**A**) In the general model for metabolosome function (2, 3), the signature enzymes are involved in the formation of the aldehyde, which is subsequently oxidized to an acyl-CoA thioester by an acylating aldehyde dehydrogenase (AldDH), or reduced to an alcohol by an alcohol dehydrogenase (ADH). The acyl-CoA is then further converted to an acyl-phosphate by a phosphotransacylase (PTA) and finally to the free carboxylic acid by a kinase (AcK), generating ATP. The NAD^+^ and CoA required for the AldDH reaction are regenerated within the BMC by the ADH and PTA, respectively. (**B**) Specific enzymes encapsulated in GRM3: The GRE-type 1,2-propanediol dehydratase and its activating enzyme (dark and light purple, respectively) represent the signature enzymes. Encapsulation peptides are depicted as small beige cylinders. The GRM gene cluster of *Rps. palustris* is shown below. Genes encoding for enzymes that are involved in the encapsulated metabolic pathway are annotated and colored: aldehyde dehydrogenase (PduP), phosphotransacylase (PduL). Genes encoding different types of shell proteins are colored in dark blue, light blue, and yellow. Ancillary genes encoding proteins and enzymes that are not directly involved in the GRM metabolic pathway are white.

In conjunction with a recent structural and functional analysis of the novel BMC-associated phosphotransacylase of *Rps. palustris*^24^ this study provides the comprehensive characterization of the catalytic core of a newly identified, widespread type of BMC. The functional characterization of the GRM3 increases the number of biochemically characterized BMCs to five and shows that GRMs also conform to the metabolic paradigm for catabolic BMCs^2^,^7^ (Fig. 1). The *in vitro* reconstitution of the central reaction sequence of the metabolic pathway of the GRM3 of *Rps. palustris* established its function in 1,2-propanediol degradation (Fig. 1B), the same overall reactions as in the canonical PDU-BMC. This represents the only known example of functional redundancy among BMCs that employ distinctly different signature (aldehyde-generating) enzymes. To our knowledge, gene clusters encoding the GRM3 or PDU-BMC never co-occur in any genome^7^, underscoring their redundant functions. The fact that some organisms employ the PDU-BMC and others use the GRM3 for propanediol metabolism has led to the speculation that the mutually exclusive use of these two strategies might reflect different ecological niches occupied. In environments that are nutrient (e.g. cobalt) limited or energy restricted, the use of the GRMs over their B_12_-dependent counterparts might be favored^7^.

Organisms that employ propanediol-utilizing GRMs comprise environmentally diverse microbes and pathogens, including *Clostridiales, Enterobacteriales, Vibrionales, Rhodospirillales, Rhodobacterales*, as well as *Rhizobiales*^7^, to which *Rps. palustris* belongs. It dwells in terrestrial and marine habitats and is able to live in anoxic sediments, where it likely uses 1,2-propanediol derived from the degradation of the plant and algal sugars fucose and rhamnose^7^. While *Rps. palustris* does not encode a conventional fuculose phosphate aldolase, it may use a remote ortholog or be dependent on other organisms for the supply of propanediol^7^. Interestingly, genomes of at least eight different strains (GenBank) of *Rps. palustris* have been sequenced, however, only BisB18 harbors the gene cluster encoding the BMC. Thus, the strain likely acquired the necessary genes in a relatively recent horizontal gene transfer event. This is also likely for other organisms. For example Some *E. coli* strains harbor a gene cluster encoding the canonical B_12_-dependent PDU BMC, whereas others encode the GRE-associated BMC, or neither. Indeed, BMC gene clusters appear to be very mobile within and across phyla^1^. Almost all of the *E. coli* strains that are able to employ one of these two types of BMCs are known pathogens^31^, indicating that both may contribute to pathogenicity and virulence in *Enterobacteriales* as has been suggested for other BMCs^32^. Therefore, components of these BMCs could be potential targets for antibiotic development.

Notably, the GRE characterized in this study shares a conserved active site architecture with GREs of the GRM5 subclass^7^. This close relationship is evidence of similar functions for GRM3 and GRM5. Besides GRE, AE and AldDH homologs, GRM5 additionally employs a canonical fuculose phosphate aldolase, as well as a putative lactaldehyde reductase^6^,^7^. Our data supports the proposed 1,2-propanediol metabolism of GRM5, in which the GRE converts propanediol produced by the sequential reactions of fuculose phosphate aldolase and lactaldehyde reductase. The propionaldehyde, is then further metabolized in the same way as in the canonical PDU-BMC and the GRM3^6^.

**Figure 2:**
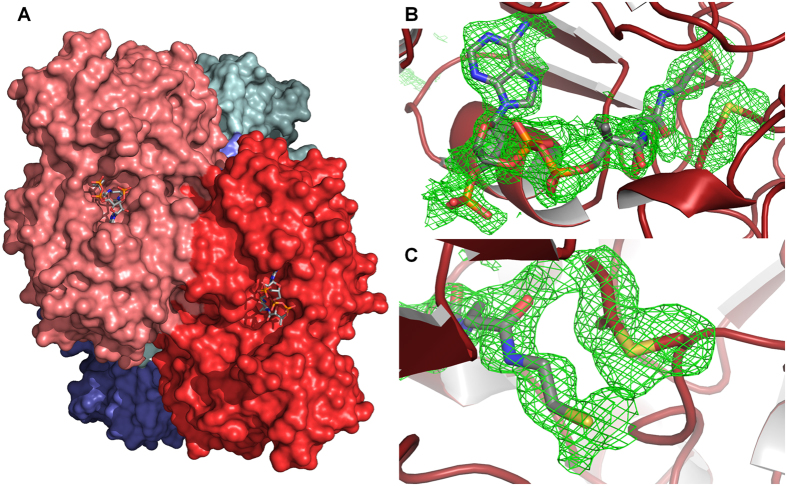
The AldDH structure (PDB 5JFN) with bound CoA and acylated Cys330. (**A**) Surface representation of the *Rps. palustris* AldDH, which forms a tetramer (dimer of dimers). A top view of a dimer interface with CoA molecules bound in the active site clefts is shown. (**B**) Superposition of an Fo-Fc electron density simulated annealing omit map at 1.8 σ (green mesh) on refined CoA and S-propionylcysteine (Cys330) in the active site of the AldDH structure. (**C**) Close-up (from ***B***) of the acylated Cys330.

**Figure 3:**
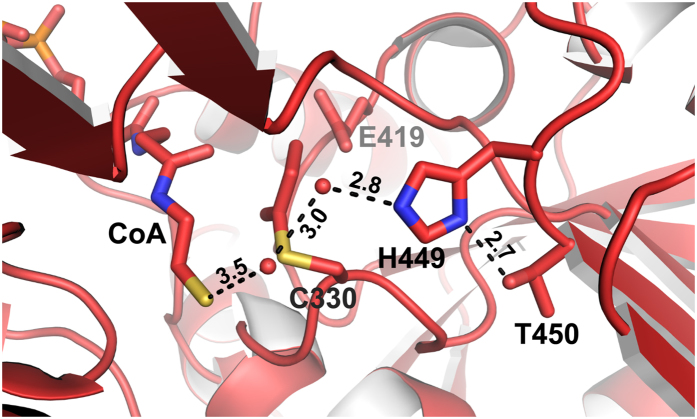
Representation of the active site of the acylating propionalydehyde dehydrogenase with bound CoA and S-propionylcysteine. Depicted is the proposed proton transfer relay, comprising Thr450, His449 as well as two structurally conserved water molecules, likely responsible for the deprotonation of CoA. Numbers indicate distances in Å.

**Figure 4:**
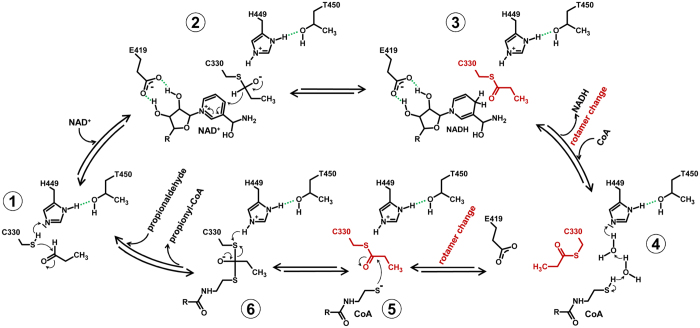
Schematic of the proposed reaction mechanism for the acylating propionyaldehyde dehydrogenase. Important hydrogen bonds are depicted in green. The switching between two different rotamer conformations of the acylated Cys330 is highlighted in red.

**Figure 5:**
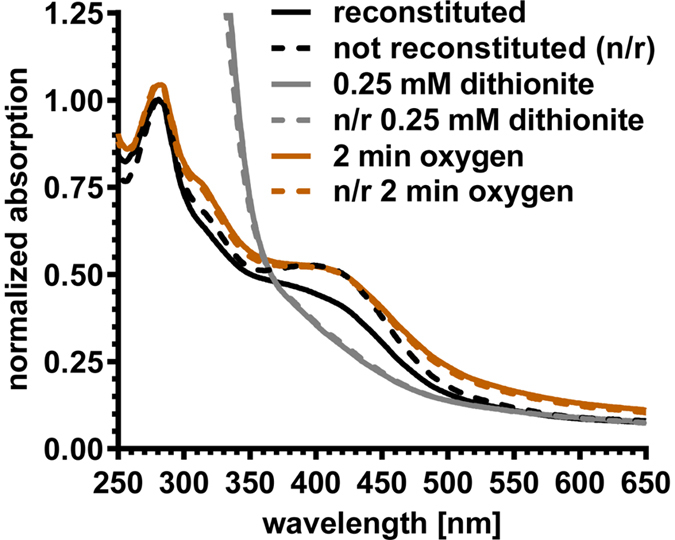
UV-VIS spectra of the [Fe-S] cluster containing activating enzyme. Reconstituted (solid lines) and non-reconstituted (broken lines) samples of the activating enzyme exhibited identical spectra in the completely dithionite reduced forms (grey), as well as in the re-oxidized form after exposure to ambient oxygen (orange). The reconstituted sample (black solid line) differs from the as prepared sample (broken black line) due to partial reduction with DTT. The spectra were normalized to the absorbance at 280 nm of the reconstituted and non-reconstituted as prepared samples (black lines).

**Figure 6:**
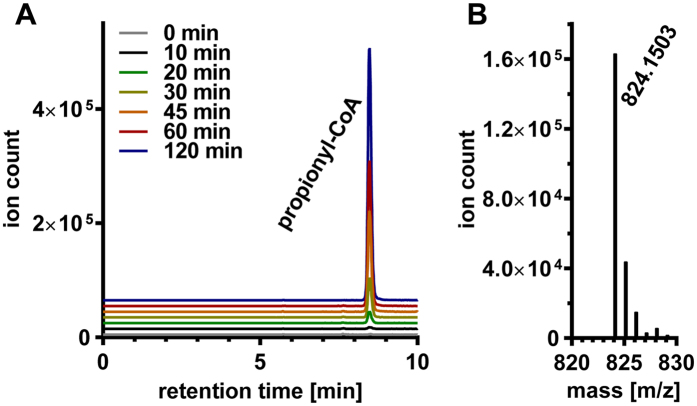
Formation of propionyl-CoA followed by HRLC-MS. (**A**) HRLC-MS extracted ion chromatogram of [M+H]+ following propionyl-CoA (calculated mass: 824.1487) formation (observed mass: 824.1503) due to the combined function of the GRE and the AldDH. (**B**) Mass-spectrum for propionyl-CoA.

The capturing of the acyl-enzyme bound thioester intermediate in an AldDH crystal structure enables us to revisit the reaction mechanism for acylating AldDHs (Fig. 4). Upon binding of the aldehyde substrate to the active site of AldDH, a conserved histidine residue (here His449) abstracts the proton of the catalytic cysteine (Cys330), which can then attack the carbonyl carbon of the aldehyde (Step 1 in Fig. 4). In the next step, NAD^+^ binds to the active site. A conserved glutamate (Glu419) forms hydrogen bonds to the nicotinamide ribose (Figs 3 and 4, Fig. S1B), which appears to facilitate the correct positioning of NAD^+^ for the hydride transfer. After NADH leaves the active site, CoA can enter and bind while the acylated cysteine residue adopts another rotamer conformation (compare Figs 2 and 3 & Step 4 in Fig. 4). When rotated away, the acylated cysteine is located between Glu419 and the thiol group of CoA, which contradicts the proposed catalytic role of the conserved glutamate in CoA thiol proton abstraction^16^. Our observation is supported by two additional observations. First, mutation of a corresponding aspartate residue to an alanine in an AldDH from *Burkholderia xenovorans* did not abolish the enzyme’s activity^33^. Second, the modeling of the CoA pantotheine moiety in the *C. phytofermentans* AldDH structure was speculative due the lack of electron density, which was pointed out by the authors^16^. Instead, we propose that His449 is also involved in the proton abstraction from CoA via two water molecules (water molecules 809 and 815 for the active site of chain B/5JFN, Fig. 3, Step 4 in Fig. 4). The rotamer switch of the cysteine provides the space for the water relay. Note that corresponding water molecules are also present in the *C. phytofermentans* AldDH structure with the bound CoA (PDB 5DBV), further evidence for a functional role. Hydrogen bonding between Thr450 and His449 ensures the correct tautomeric form for proton abstraction from the cysteine as well as from the first water of the proton relay (Figs 3 and 4). Supported by the position of the propionyl moiety in the structure with bound propionyl-CoA (Fig. S1A), the acylated cysteine would revert into the first rotamer conformation to be attacked by the activated CoA thiol, facilitating the acyl-CoA formation (Step 5 & 6 in Fig. 4) and completing the reaction cycle. In summary, characterization of the GRM3 AldDH leads to a revised and detailed reaction mechanism for the ubiquitous (present in all three domains of life) family of acylating aldehyde dehydrogenases. These enzymes take part in numerous pathways including fermentations, detoxifications, other catabolic and central carbon metabolic processes. Our findings apply beyond BMC function and will be of value for the bioengineering of these enzymes towards different substrates, particularily (toxic) aldehydes that may be formed in novel synthetic pathways for the microbial production of value-added chemicals^34^.

The addition of yet another function to the catalytic repertoire of GREs (propanediol dehydratases) in this as well as another recent study^13^ underscores the versatility of this ancient and widespread enzyme family. Our findings demonstrate that GREs associated with BMCs can be divided into (at least two different types) choline TMA-lyases and 1,2-propanediol dehydratases. The apparent versatility of GREs is useful for understanding their potential roles in diverse microbes and for bioengineers striving to co-opt BMCs and particularly GRMs to fulfill novel, designed functions. A BMC shell may confer additional protection against molecular oxygen or reactive oxygen species for the extremely sensitive GREs and AEs and thereby extend the range of habitats wherein they may be useful to microbes. It is also important to note that the AldDH characterized in this study exhibits much higher rates of catalysis (~2 to 3 orders of magnitude) than other previously characterized orthologs^16^,^34^,^35^, and is able to accept not only acetyl-CoA but also 3-hydroxypropionyl-CoA, which is of interest to bioengineers aiming to produce 3-hydroxypropionate (a high value chemical building block) from glycerol 34.

In addition to providing fundamental insights into the physiological function of GRMs, the structural and functional characterization presented here sheds light on novel aspects of the assembly and organization of the enzymatic core of the GRM3. It appears that the AldDH as well as the GRE contain novel types of EPs, which comprise two consecutive α-helices. The exact mode of function of these EPs needs to be investigated further, as the putative intrinsic inter-domain EP of the GRM3 type GRE represents an intriguing and unprecedented example of an intra-protein EP and suggests that determinants for assembly into BMCs could be engineered between protein domains. The removal of the putative inter-domain EP resulted in production of an increased amount of soluble and yet catalytically active enzyme. GRM3 and perhaps other GRMs may assemble in an ‘inside-out’ fashion, where (some) of the encapsulated enzymes aggregate first, before the shell proteins are recruited^2^,^15^,^36^,^37^. The interaction between the AE and the GRE is likely responsible for the recruitment of the AE during BMC formation. In contrast, the EP of the AldDH did not lead to the aggregation of the enzyme, suggesting it may only interact with shell proteins. These results are consistent with the growing evidence that EPs can be involved in both core assembly and recruitment of the shell^15^,^24^,^36^. Collectively these observations are not only important for the fundamental understanding of BMC assembly, but also inform the design and construction of custom nanoreactors in synthetic biology^38^,^39^.

## Methods

### Chemicals

Chemicals were purchased from Carl Roth and Sigma-Aldrich. Acetyl-CoA and propionyl-CoA were synthesized and purified from their respective carboxylic acid anhydrides^40^. 3-hydroxypropionyl-CoA was synthesized and purified from a mixed anhydride using carbonyldiimidazole as coupling agent^40^.

### Cloning of the aldehyde dehydrogenase, the glycyl radical enzyme, and the activating enzyme from *Rps. palustris* BisB18

We performed BLAST searches for each of the enzymes encoded in the BMC gene cluster of *Rps. palustris* BisB18. For AldDH, the aldehyde dehydrogenase, we noted that the primary structures of closely related homologs were about 50 residues longer, suggesting the start codon was mis-assigned in the GRM locus. Indeed, we found another start codon further up-stream, as well as an apparent Shine-Dalgarno sequence (AAGGAG) separated by 7 base pairs from that start codon. Instead of 464 amino acids (as in the GenBank record) the enzyme encoded by the revised full-length gene consisted of 512 amino acids.

The genes coding for the GRE, AE, and AldDH from *Rps. palustris* BisB18 were amplified using chromosomal DNA as the template. Standard PCRs were performed with Phusion^®^ polymerase (ThermoFisher). The PCR products were cloned into vectors for expression in *Escherichia coli*. Used primers and the resulting plasmid constructs are listed in Table S1.

### Heterologous expression of the acylating aldehyde dehydrogenase (AldDH)

*E. coli* BL21 (DE3) cells containing the expression plasmid (pPduP_Rp_JZ73) for a Strep-tagged AldDH were grown in LB-medium. Ampicillin was added to cultures to concentrations of 100 μg/ml. Due to leaky expression, the cultures were grown for 24 h at 25 °C without IPTG induction.

### Heterologous expression of the glycyl radical enzyme and its activating enzyme

Cultures of *E. coli* BL21 (DE3) were grown in a MOPS/NaOH buffered (100 mM, pH 7.5) LB-medium. First, bottles with MOPS buffer were made anaerobic by sparging with nitrogen gas. The bottles were capped and transferred into an anaerobic glove box. The buffer was then supplemented with LB-broth (EMD Millipore). The bottles were sealed with rubber stoppers and autoclaved. The sterile media bottles were then anaerobically supplemented with 25 mM glucose, 25 mM sodium fumarate, and 100 μg/mL ampicillin from sterile stock solutions. For the activating enzyme, the medium was also supplemented with 1 mM L-cysteine, 1 mM ferric ammonium citrate. The bottles were then inoculated with 10 ml of an aerobically grown pre-culture of cells containing the expression plasmids for the glycyl radical enzyme or the activating enzyme (pGRE_Rp_JZ15, pGRE_Rp_no-EP_JZ88, or pGRE-AE_Rp_JZ16). The anaerobic cultures were grown at 30 °C with agitation (120 rpm). When the cultures reached an OD_600nm_ of 1.0 the cultures were induced with 0.4 mM IPTG and grown for another 16 hours. The cells were harvested under anaerobic conditions.

### Purification of the aldehyde dehydrogenase (AldDH)

Cells were resuspended in a 3-fold volume of 50 mM MOPS/KOH buffer (pH 7.5) containing 150 mM KCl (buffer A) with 0.1 mg DNase I per ml. The cells were lysed by passage through a chilled French pressure cell at 137 MPa. The cell lysates were ultracentrifuged for 1 h at 100,000 × *g* at 4 °C and the supernatants were applied at a flow rate of 0.5 ml min^−1^ to a 1 ml StrepTrap column (GE Healthcare) that had been equilibrated with buffer A. The column was washed with buffer A and the recombinant enzyme was eluted with the same buffer A with the addition of 2.5 mM desthiobiotin. Fractions containing AldDH activity were pooled and concentrated using a Amicon Ultra-4 Centrifugal Filter Unit (EMD Millipore) with a 30 kDa pore size. The sample was then applied to a 120 ml gel filtration column (HiLoad 16/600 Superdex 200 pg, GE Healthcare), equilibrated with 20 mM MOPS/KOH pH 7.5 buffer containing 50 mM KCl.

To render the enyzme anaerobic, it was applied to a 5 mL HiTrap Desalting column (GE Healthcare) that had been equilibrated with 100 mM MOPS/KOH at pH 7.5 containing 150 mM KCl under anaerobic conditions.

### Purification of the glycyl radical enzyme and its activating enzyme

Cells were resuspended in a 3-fold volume of 50 mM MOPS/KOH at pH 7.5 containing 250 mM KCl (buffer B) with 0.1 mg DNase I per mL and lysed using a French press (137 MPa) under anaerobic conditions. The lysates were centrifuged for one hour at 100,000 × *g* and 4 °C. All purification steps were performed in an anaerobic glove box. The 100,000 × *g* supernatants were applied to a 1 mL HisTrap HP column (GE Healthcare) that was equilibrated with buffer A. After loading of the sample the column was washed with buffer A.

For purification of the GRE the column was additionally washed with 80 mM imidazole in buffer B to remove unspecifically bound proteins. The enzyme was eluted with 500 mM imidazole in buffer A.

For the AE, the column was washed with 50 mM imidazole in buffer B to remove unwanted protein. The activating enzyme slowly eluted at the same concentration of imidazole in a second very broad peak.

To co-purify the GRE and AE, the harvested cell pellets were combined prior lysis and treated as above. After loading the sample to the HisTrap-column it was washed with 60 mM imidazole in buffer B. The GRE and AE co-eluted at 500 mM imidazole in buffer B.

Both the GRE and AE or the mixtures were concentrated using Amicon Ultra-4 Centrifugal Filter Units (EMD Millipore) with 10 kDa pore sizes. To remove the imidazole the enzymes were applied to a desalting column (HiTrap Desalting 5 mL, GE Healthcare) that had been equilibrated with 100 mM MOPS/KOH at pH 7.5 containing 150 mM KCl.

### Iron-sulfur cluster reconstitution for the activating enzyme

After nickel affinity chromatography, half of the AE sample was incubated under anaerobic conditions with 5 mM DTT, 0.5 mM ammonium iron(II) sulfate, and 0.5 mM sodium sulfide for 12 h at 4 °C. To remove excess iron salts both the treated and untreated AE samples were applied to a HiLoad 16/600 Superdex 75 pg (GE Healthcare) gelfiltration column equilibrated with a 50 mM MOPS/KOH buffer (pH 7.5) containg 150 mM KCl. The purified proteins were then stored anaerobically at 4 °C until further use for UV-VIS photospectroscopy.

### UV-VIS photospectroscopy of the activating enzyme

UV-VIS spectra of reconstituted and non-reconstituted AE were recorded anaerobically in rubber sealed quartz cuvettes using an Agilent Cary 60 spectrophotometer. Samples of reconstituted and non-reconstituted AE (in 50 mM MOPS/KOH buffer, pH 7.5, 150 mM KCl) were mixed with 2 mM DTT or 0.5 mM sodium dithionite and incubated for 2 min before recording the spectra. UV-VIS spectra were also recorded after exposing the AE samples to oxygen.

### Crystallization and structure determination of AldDH

For co-crystallization of the purified Strep-tagged AldDH in the presence of propionyl-CoA two different conditions were used. *1*) AldDH (2.4 mg mL^−1^) was mixed with 0.1 M HEPES/NaOH pH 7.0, 19% (w/v) PEG 4000, and 4 mM propionyl-CoA in a ratio of 2 μL:3 μL (enzyme to crystallization buffer). *2*) AldDH (6 mg mL^−1^) was mixed with 50 μM sodium citrate pH 4.8, 4% (w/v) PEG 8000, and 5 mM propionyl-CoA in a ratio of 3 μL:6 μL (enzyme to crystallization buffer).

For co-crystallization in the presence of NAD^+^ Strep-tagged AldDH (7.5 mg mL^−1^) was mixed with 50 μM sodium citrate pH 4.8, 4% (w/v) PEG 8000, and 5 mM NAD^+^ in a ratio of 3 μL:6 μL (enzyme to crystallization buffer).

Crystals were soaked briefly with mother liquor supplemented with 35% (v/v) PEG 400, and 5–10 mM of propionyl-CoA or NAD^+^, respectively, before freezing in liquid nitrogen. X-ray diffraction data were collected at the Advanced Light Source (beamlines 5.0.2, 5.0.3) at Lawrence Berkeley National Laboratory.

The data was processed with the XDS software package^41^. The structures were solved by molecular replacement using the structure of a propionaldehyde dehydrogenase from *Clostridium phytofermentans*^16^ (PDB 4C3S) as the search model. The molecular replacement was carried out using PhaserMR of the Phenix software package^42^. Initial models were built with Phenix.AutoBuild and refined with the phenix.refine. Additional modeling, manual refining and ligand fitting was done in COOT^43^. Final positional and B-factor refinements, as well as water-picking for the structures, were performed using phenix.refine.

### AldDH activity assay

The activity of AldDH was routinely measured in photometric assays in the forward and reverse directions. For the forward direction the assay mixture (0.4 mL) contained 200 mM MOPS/KOH (pH 7.5), 5 mM NAD^+^, 0.5 mM CoA, and purified AldDH. The reaction was initiated by addition of propionaldehyde or acetaldehyde. The aldehyde and CoA- dependent reduction of NAD^+^ was monitored at 340 nm. After completion of the reaction, 100 mL were withdrawn and enzyme was precipitated by addition of 5 μL 98% (v/v) formic acid. The precipitate was removed by centrifugation at 17,000 × *g* and 4 °C. The supernatants were analyzed by HRLC-MS to confirm the production of acetyl-CoA or propionyl-CoA, respectively.

For the reverse direction, the assay mixture (0.4 mL) contained 200 mM MOPS/KOH (pH 7.5), 0.4 mM NADH, 0.25 mM CoA-thioester (acetyl-CoA, propionyl-CoA or 3-hydroxypropionyl-CoA), and purified AldDH. The CoA-thioester dependent oxidation of NADH was monitored at 365 nm.

### Enzyme activity assay for the glycyl radical enzyme under anaerobic conditions

Co-purified truncated GRE and AE was incubated in 100 mM MOPS/KOH at pH 7.5 containing 150 mM KCl (final volume of 400 μL) in the presence of 50 mM (*R*/*S*)-1,2-propanediol (or 50 mM glycerol), 0.5 mM sodium dithionite, 1 mM SAM, 0.5 mM CoA, 2.5 mM NAD^+^, and anaerobic propionaldehyde dehydrogenase (AldDH). The reaction mixture was incubated at 16 °C for 2 hours. Samples of 50 μL were withdrawn after 0, 10, 20, 30, 45, 60, and 120 min. The reactions were stopped by addition of 5 μl of 98% (v/v) formic acid. Precipitated protein was removed by centrifugation at 17,000 × *g* and 4 °C. The supernatants were analyzed for the presence of propionyl-CoA, 3-hydroxypropionyl-CoA, and SAM cleavage products by HRLC-MS.

### High Resolution Liquid Chromatography-Mass Spectrography (HRLC-MS)

CoA, propionyl-CoA, and 3-hydroxypropionyl-CoA, SAM, MTA, and dA were analyzed using an Agilent 6550 iFunnel Q-TOF LC-MS system equipped with an electrospray ionization source set to positive ionization mode.

Compounds were separated on a RP-18 column (50 mm × 2.1 mm, particle size 1.7 μm, Kinetex XB-C18, Phenomenex) using a mobile phase system comprised of 50 mM ammonium formate pH 8.1 and methanol. Chromatographic separation was carried out using the following gradient condition at a flow rate of 250 μl/min: 0 min 0% methanol; 1 min 0% methanol, 3 min 2.5% methanol; 9 min 23% methanol; 14 min 80% methanol; 16 min 80% methanol.

Capillary voltage was set at 3.5 kV and nitrogen gas was used for nebulizing (20 psig), drying (13 l/min, 225 °C) and sheath gas (12 l/min, 400 °C). The TOF was calibrated using an ESI-L Low Concentration Tuning Mix (Agilent) before measurement (residuals less than 2 ppm for five reference ions) and was recalibrated during a run using 922 m/z as reference mass. MS data were acquired with a scan range of 200–1200 m/z.

LC-MS data were analyzed using MassHunter Qualitative Analysis software (Agilent).

## Additional Information

**How to cite this article**: Zarzycki, J. *et al. In Vitro* Characterization and Concerted Function of Three Core Enzymes of a Glycyl Radical Enzyme - Associated Bacterial Microcompartment. *Sci. Rep.* **7**, 42757; doi: 10.1038/srep42757 (2017).

**Publisher's note:** Springer Nature remains neutral with regard to jurisdictional claims in published maps and institutional affiliations.

## Supplementary Material

Supplemental Information

## Figures and Tables

**Table 1:** Data collection and refinement statistics for AldDH crystal structures.

Ligands	CoA and S-propionylcysteine	Propionyl-CoA	NAD^+^
PDB ID	5JFN	5JFM	5JFL
Space group (No)	*P* 1 2_1_ 1 (4)	*P* 1 (1)	*P* 1 2_1_ 1 (4)
Cell dimensions			
* a b c* [Å]	83.19 106.45 125.73	92.11 105.78 126.43	107.39 154.80 126.59
* α β γ* [°]	90.00 109.03 90.00	89.52 71.00 68.92	90.00 90.13 90.00
Resolution [Å]	39.2–1.90 (2.00–1.90)	39.26–2.52 (2.65–2.52)	33.64–2.30 (2.42–2.30)
Number of observations			
* *total	716,571	308,204	1,228,225
* *Unique	162,389	135,429	181,023
Redundancy	4.4 (4.4)	2.3 (2.2)	7.1 (6.8)
*I*/σI*	12.8 (2.1)	11.3 (2.3)	10.9 (2.7)
Completeness [%]	99.7 (98.3)	95.5 (91.8)	98.9 (98.0)
Refinement			
*R*_work_/*R*_free_	16.2/18.7	17.2/22.3	18.9/22.9
No. atoms	15,377	28,030	27,656
* *Protein	13,245	26,527	26,386
* *Ligand/ion	348	412	352
* *Water	1784	1091	827
Average B-factors	22.1	37.5	38.9
* *Protein	20.1	37.1	39.0
* *Ligand/ion	44.7	64.8	45.3
* *Water	33.1	36.3	35.6
R.m.s deviations			
* *Bond lengths [Å]	0.004	0.006	0.004
* *Bond angles [°]	0.69	0.78	0.62
Ramachandran			
* *favored	98.9	98.5	96.4
* *allowed	1.10	1.45	3.50
* *outliers	0.00	0.06	0.14

Numbers in parentheses indicate statistics for highest resolution shell.

**Table 2:** Catalytic properties of the recombinant aldehyde dehydrogenase from *Rps. palustris* (AldDH).

	*V*_max_ [μmol min^−1^ mg^−1^]	*k*_cat_ [s^−1^]*	apparent *K*_M_ [mM]	*k*_cat_/*K*_M_ [M^−1^ s^−1^]*
acetaldehyde	117 ± 8	433	4.1 ± 0.7	105.6 × 10^3^
propionaldehyde	242 ± 14	895	1.2 ± 0.17	745.8 × 10^3^
acetyl-CoA^†^	5.3 ± 0.3	19.6 ± 1.1	n.d.	n.d.
propionyl-CoA^†^	31.6 ± 1.9	116.9 ± 7.0	n.d.	n.d.
3OHP -CoA^†^	2.4 ± 0.05	8.9 ± 0.2	n.d.	n.d.

^*^Per tetrameric enzyme, ^†^at 250 μM, n.d. – not determined.
