# Supplementary material for: In Vitro Characterization and Concerted Function of Three Core Enzymes of a Glycyl Radical Enzyme - Associated Bacterial Microcompartment
Source: Sci Rep. 2017 Feb 16;7:42757. doi: 10.1038/srep42757 (PMC5311937; doi:10.1038/srep42757)
Supplement: Supplemental Information [file srep42757-s1.pdf]

In Vitro Characterization and Concerted Function of Three Core Enzymes of a Glycyl Radical Enzyme - Associated Bacterial Microcompartment

Jan Zarzycki, Markus Sutter, Niña Socorro Cortina, Tobias J. Erb, Cheryl A. Kerfeld

SUPPORTING INFORMATION

Table S1. List of used primers for cloning

Primer name	Primer sequence*	Cut site	Plasmid
pduP_BisB18_for	TAT**CATATG**AATGACGCAAACATCGCCGATG	NdeI	pPduP_Rp_JZ73
pduP_BisB18_for	TAT**GAATTC**AACGGATATTGAGCGCTTCCACC	EcoRI	
GRE_Rp_for	GCTCGATC**CATATG**ATCGAAAAAGGTTTCTCCAAG	NdeI	pGRE_Rp_JZ15
GRE_Rp_rev	CCGGGACAAACCCTT**CTCGAG**CTACAAGG	XhoI	
AE_Rp_for	GGACAATC**CATATG**AGCGAGATCAAG	NdeI	pAE_Rp_JZ16
AE_Rp_rev	GCTCGATC**CTCGAG**TCCGCCGATCAGACAAC	XhoI	
GRE_noEP_for	CATA**GCTAGC**GGTGCCTCGTCGAG	NheI	pGRE_noEP_JZ88
GRE_noEP_rev	TTAT**GCTAGC**GAAATTGGCGTCGAGCG	NheI	

* Bold letters represent restriction sites.

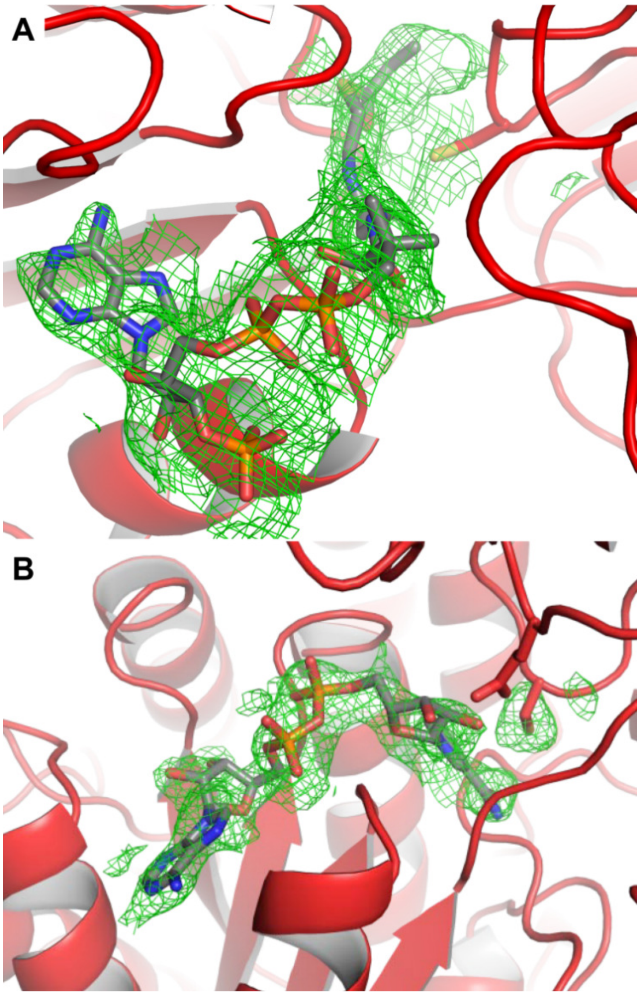

Figure S1. Simulated annealing Fo-Fc electron density maps at 1.8 σ of the active site region.

(A) Omit map for propionyl-CoA and Cys330 side chain atoms (PDB 5JFM). *(B)* Omit map for NAD^+ and Cys330 side chain atoms (PDB 5JFL). Glu419 coordinates the nicotineamide ribose.

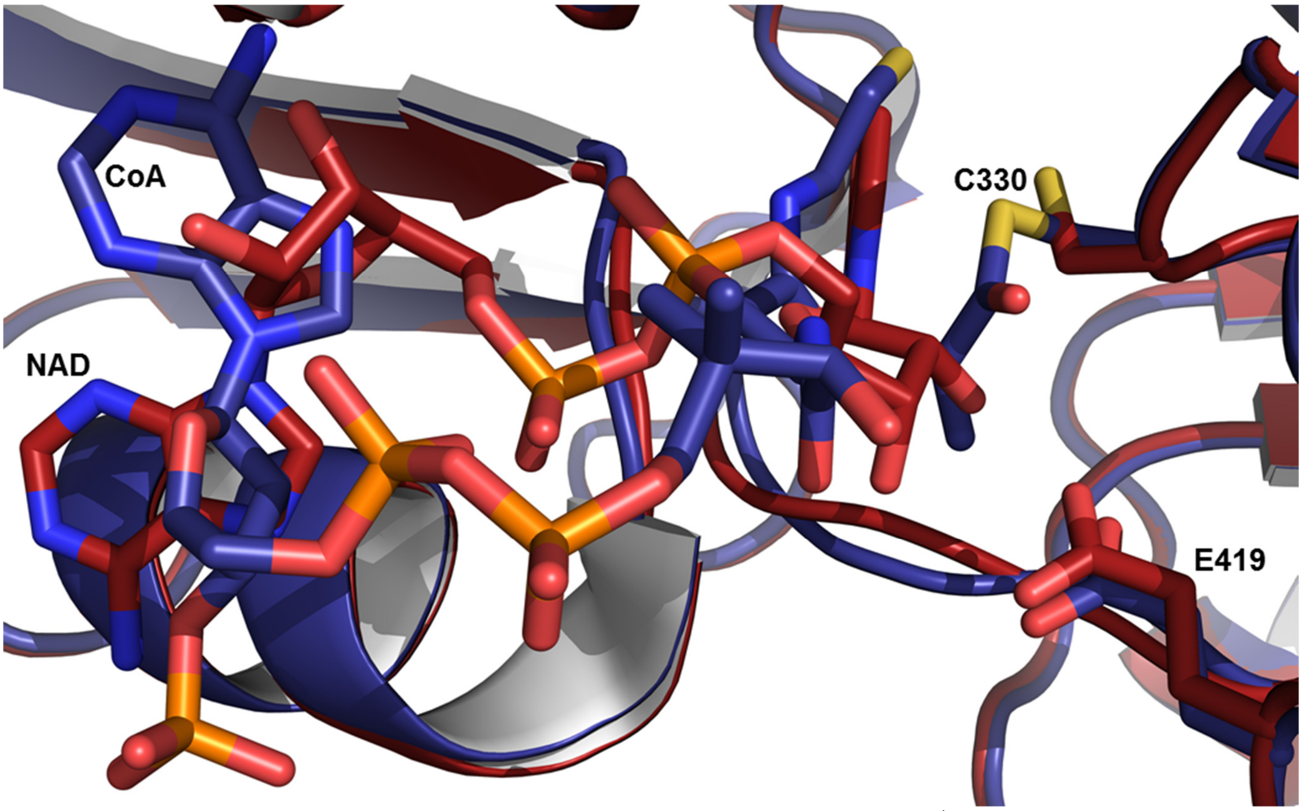

Figure S2. Superposition of the AldDH structures with bound CoA and NAD^+.

The CoA molecule in PDB 5JFN (red) occupies a similar position as the NAD^+ in PDB 5JFL (blue). The adenine moieties of both molecules are labeled and point in different directions. Also depicted are Glu419 as well as are both forms (propionylated and native) of the catalytic Cys330 residue.

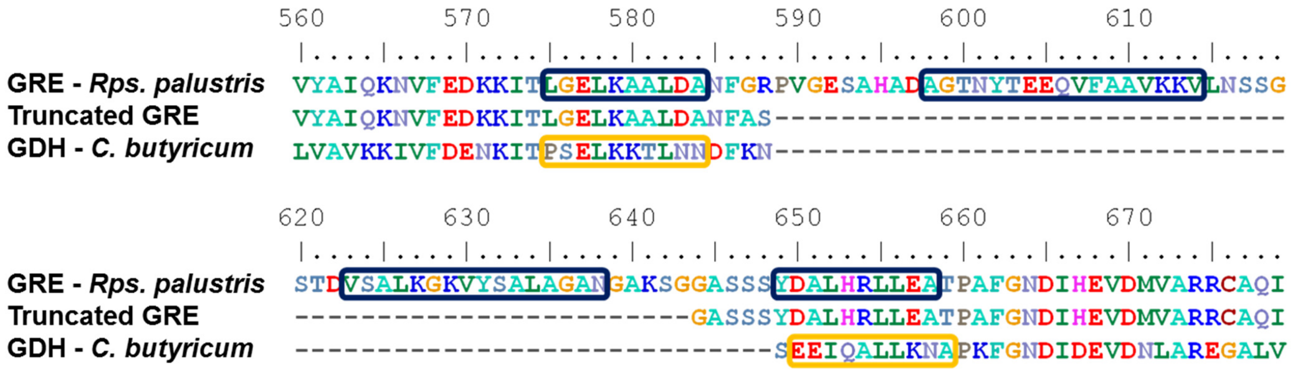

Figure S3. Amino acid sequence alignment for the glycyl radical enzyme.

Shown are the aligned central regions of the GRE from *Rps. palustris* harboring the putative encapsulation peptide. Also shown is the designed truncated version of the GRE, and the glycerol dehydratase (GDH) from *C. butyricum*. Predicted alpha-helices are highlighted in blue. Helices observed in the crystal structure (PDB 1R9D) of the GDH are highlighted in yellow. The truncated version of the *Rps. palustris* GRE is lacking only the predicted internal encapsulation peptide represented by the two central helices.

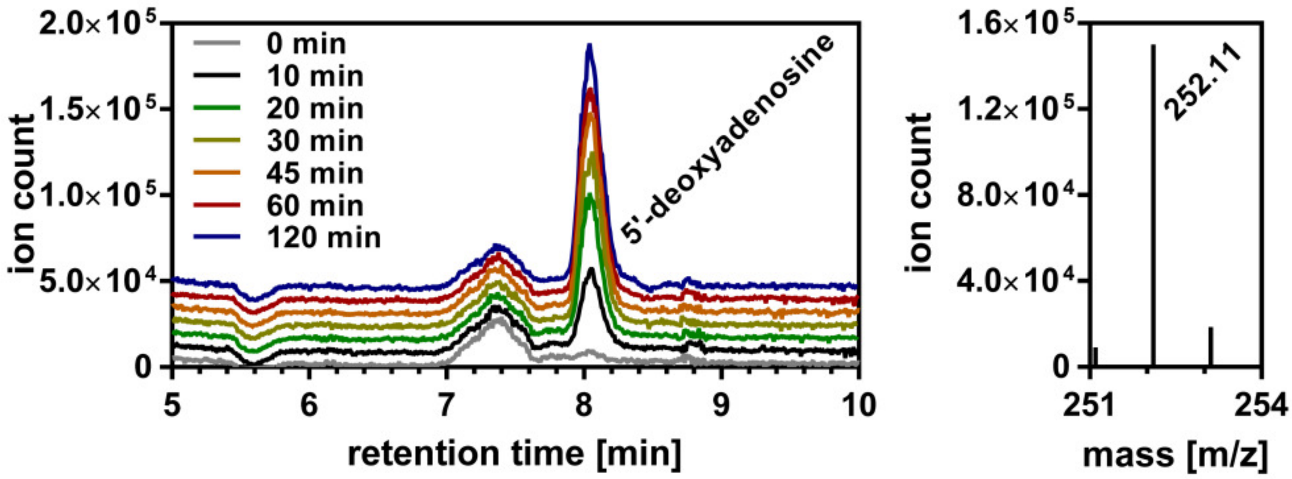

Figure S4. HRLC-MS extracted ion chromatograms (left) and mass-spectra (right) for the SAM cleavage product 5'-deoxy adenosine.

The amount of the SAM cleavage product, 5'-deoxy adenosine (calculated mass: 252.1091 $[M+H]^+$, found mass: 252.11 $[M+H]^+$), increased over time.
